# Trusting the postman: prosecuting theft and managing sickness in the British Post Office, c.1860–1910

**DOI:** 10.1080/03071022.2025.2431396

**Published:** 2025-03-04

**Authors:** David Green, Natasha Preger, Harry Smith

**Affiliations:** a King’s College London; b University of Exeter

**Keywords:** Trust, theft, sickness, Post Office, United Kingdom, nineteenth century

## Abstract

The Post Office was an immensely important institution of the British state. It fostered communication, encouraged business, provided employment and generated revenue for the Treasury. Efficiency and economy were paramount considerations for the Post Office authorities, keen to maintain the government’s trust in good management. Maintaining public trust to deliver mail and messages speedily and securely also underpinned its operations. When trust was called into question, often due to theft of mail by postal workers themselves or the rising costs of sick leave, the Post Office was keen to act. In this article we examine two critical points of tension in the Post Office that tested trust in the institution. The first relates to the incidence of mail theft by its own workers and the actions taken by the Post Office authorities to catch and prosecute the perpetrators. The second relates to the incidence of sickness and the attempts to monitor the legitimacy of claims for sick pay. Both instances lay bare the workings of the Post Office and the critical importance of trust in its operations and, more widely, in late nineteenth-century urban society.

The start of August 1875 was a quiet week for news in Britain. Parliament was in summer recess and political stories were in short supply. The newspaper columns were filled with reports of domestic and foreign matters, commercial information, tales of missionary life, freak accidents and sporting intelligence. The court reports included the usual parade of assaults, arson, theft and forgeries. The reports also included two cases related to crimes committed by postal workers.

The first case involved embezzlement by a 17-year-old postal clerk from Guildford called Arthur Dunham. It was heard at Croydon Assizes where a guilty verdict was recorded and a sentence of five years’ penal servitude imposed – a relatively harsh sentence clearly designed to act as a deterrent to other youthful employees who might be tempted to steal from the Post Office.^1^^1^*Epsom Journal*, 27 July 1875, 2; *Morning Post*, 30 July 1875, 8. Penal servitude was considered to be a more severe form of punishment than hard labour. The second case was that of Frank Foley, aged 25, who worked as an auxiliary letter carrier in East London, charged at Worship Street Police Court with being drunk on his round and of damaging the cell in which he was held after being arrested. Foley’s responsibilities were to collect the letters from the post boxes and receiving houses and to return them to the post office where he was employed. At about 5.30pm on Wednesday 5 August, while still in uniform, Foley was found drunk with a bag full of undelivered letters. Foley claimed that he had taken a quantity of brandy to cure a bout of diarrhoea – a plea that did not convince Mr Bushby, the magistrate, who imposed a fine of £10 or two months in prison, and a further 15 shillings or seven days in prison for damaging the cell. Mr Bushby’s main source of displeasure with Foley’s actions rested not on the damage he had caused but on the abuse of his position of responsibility as a postman while still in uniform. The court reporter concluded: ‘Mr Bushby said that a postman who got drunk on duty committed a most serious offence, as the letters in his care might contain valuable property, and, besides, he was in trust of, possibly, the happiness of whole families’.^2^^2^*East London Observer*, 7 August 1875, 2.

The crimes committed by Dunham and Foley were, in themselves, relatively minor but they spoke to a wider set of issues that were of crucial concern not just for the Post Office but for the state itself. Dunham had stolen money from the Post Office and Foley had failed to deliver his mail on time on account of being drunk, but more significantly both had abused the trust placed in them as officers of the state, and this was the central issue that lay at the heart of their convictions. Trust that workers would perform their duties with ‘diligence and fidelity’ – terms that were written into the contract of employment for civil servants, including postal workers – were crucial to ensure public confidence that the mail would be delivered securely and efficiently. Trust was made even more important as the Post Office expanded its activities to include the Savings Bank, which replaced the Money Order Office in 1861, and its increasing involvement in transmitting money through the mail by postal orders, which were introduced in 1881. Its growing importance in financial transactions tied the Post Office inextricably to the growth of the British economy and therefore the state itself. According to Patrick Joyce, the two institutions were inextricably linked, for ‘In systematising the communication of words, and numbers, it [the postal service] enabled the development and the co-ordination of all the other infrastructural domains upon which the state increasingly rested’.^3^^3^P. Joyce, *The State of Freedom* (Cambridge, 2013), 54.

In addition to its cultural and political importance, however, the Post Office was also a very visible extension of the state into the everyday lives of its citizens. It became by far the largest employer in the country, with a workforce close to 167,000 by the end of the century, based in nearly 22,000 post offices distributed across the entire kingdom.^4^^4^M. Daunton, *Royal Mail: The Post Office since* 1840 (London, 1985), 194, 276. The pillar box, introduced in 1853 and from 1874 painted in red, together with the uniformed postman, who was the official with whom the public came into contact most frequently, were physical embodiments of the state itself and signifiers of its qualities.

In addition, the Post Office was an important revenue-producing department of the state, contributing significant amounts to the Treasury each year.^5^^5^See also Joyce, *op. cit*., 111–17. In 1860 it had a turnover of over £3.5 million which, after taking costs into account, amounted to a profit of over £700,000. By the end of the century these figures had risen fivefold to over £16.8 million and £3.4 million, respectively.^6^^6^These figures come from the annual reports of the Postmaster General which are published in the British Parliamentary Papers (subsequently BPP). See 16th (1860) and 46th (1900) annual reports of the Postmaster General. The growing financial importance of the Post Office for the Treasury is reflected in its annual share of government income: the annual turnover figures represented around 5% of the total gross public income in 1860, rising to over 13% by 1900.^7^^7^Figures for gross public income are derived from B.R. Mitchell and P. Deane, *Abstract of British Historical Statistics* (London, 1962), 393–94. The financial success of the Post Office’s operations was paraded annually in the Postmaster General’s reports, and the Treasury took a keen interest in its operations, not least because the running costs, including wages, sick pay, gratuities and pensions, came from its coffers. Governments therefore kept a watchful eye over the Post Office, keen to ensure that nothing undermined public confidence or interfered with its ability to return a profit. In return, Post Office officials were acutely aware of their fiscal responsibilities and keen to maintain a reputation for efficient and economical management.^8^^8^C. Perry, *The Victorian Post Office: The growth of a bureaucracy* (Woodbridge, 1992), 30–49. In that context maintaining the government’s trust, as well as that of the public, was of critical importance to the Post Office authorities. It was a serious matter whenever this was called into question and prompted measures and responses by the Post Office to ensure that trust between workers and the public, and between the organisation itself and the Treasury, remained intact.

What happens, therefore, when those measures and responses are called into question? What are the consequences associated with failing to deliver the mail or with workers who feigned sickness and took unauthorised leave? It is at these critical disjunctures that, as Patrick Joyce puts it, ‘the material and human performance of predictability, reliability and dependency’ is called into question.^9^^9^Joyce, *op. cit*., 122–23. These disjunctures are the focus of this article. We concentrate on two points of tension when trust between the public and the Post Office, and within the organisation itself, was questioned. We explore, first, the issue of public confidence associated with the theft of mail and the efforts made to identify and prosecute culprits, arguing that the public nature of such efforts was crucial in helping to reinforce trust in the institution itself. It was not just that the culprits were caught and punished but that this was done in a public and performative way, as the newspaper reports of Dunham and Foley’s misdemeanours, and those of many other postal workers whose crimes were reported in the press, demonstrated. In the second part we explore the complex issues of trust and responsibility that emerged between the Post Office and the state relating to the arrangements for sick leave and pensions. The relatively generous levels of pensions and sick pay available to postal workers raised issues about workers’ ability to perform their roles over a lifetime of service. It also raised questions about personal behaviour – notably individual responsibility for maintaining good health and the temptation to malinger presented by the availability of sick pay. To ensure that only healthy workers were employed and to counter the risk of malingering, which was of constant concern to the Post Office, a raft of measures was introduced, including the creation of a medical service and the employment of doctors who were responsible for ensuring that only fit workers were taken on and for monitoring and certifying sick leave. Taken together, the issues surrounding the problems of crime and sickness help to illuminate the importance of trust in the Post Office during a crucial period of growth.^10^^10^For general histories of the Post Office during this period see A. Clinton, *Post Office Workers: A trade union and social history* (London, 1984); Daunton, *op. cit*.; Perry, *op. cit*.; D. Campbell-Smith, *Masters of the Post* (London, 2012).

## Stealing the mail

**Figure 1.**
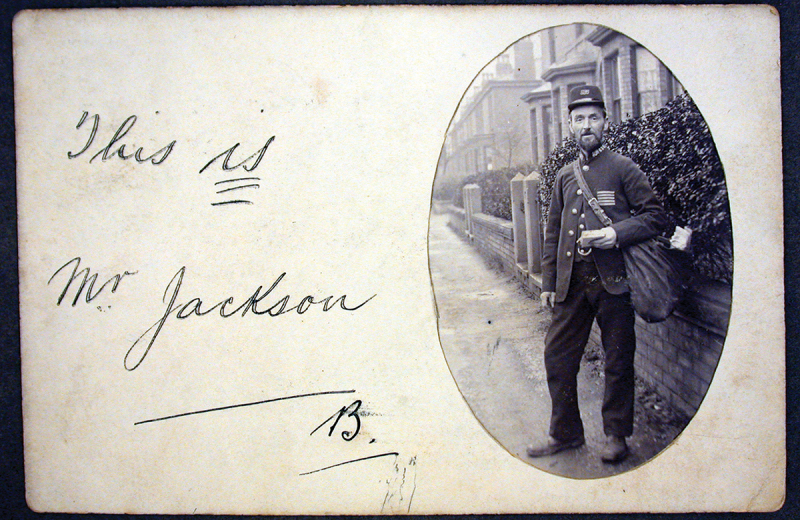
‘This is Mr Jackson’, c.1908.

Source: The Postal Museum collection, 2005–0082/50. Reproduced with kind permission of The Postal Museum. The five stripes sewn onto the uniform represented at least 25 years’ service in the Post Office.

Postal workers were often familiar and trusted figures in their local communities. They interacted frequently with the public as postmasters and postmistresses and postmen and postwomen, conveying through the mail not only money and valuables but also the most intimate of messages. Uniforms, rules, and expectations of moral rectitude, together with the efforts made to deliver the mail under sometimes difficult circumstances, helped to reinforce the view that above all the Post Office and its workers could be trusted. The materiality of that trust was manifested in the postman’s uniform. Those responsible for delivering the mail, in contrast to indoor workers such as clerks and sorters, were distinguished by their uniforms as loyal employees of the state, and those who had an unblemished service record extending over a period of years were awarded extra pay and good conduct stripes which were prominently displayed on the front of their jacket (see Figure 1).^11^^11^Good service stripes were awarded after five years’ unblemished service and involved an additional one shilling a week extra pay. Up to 1896 postmen could accrue up to three stripes; from the following year this was raised to six. See Postmaster General’s 43rd Annual Report (1897), 28. In 1898, following the liberalisation of the award of stripes, 17,282 postmen held from one to six stripes, representing approximately half of all established postmen and one in five members of the total workforce. See Postmaster General’s 44th Annual Report (1898), 28.

As servants of the state, Post Office workers were required to conform to a wide range of rules that governed their behaviour at work and clear expectations of moral rectitude beyond the confines of the job. They were expected to serve with ‘diligence and fidelity’, qualities which ultimately were required in order to receive a pension. Misdemeanours, such as entering a public house or accepting drink while in uniform, were treated as disciplinary offences, and culprits were likely to be fined, demoted or in the more serious cases, suspended.^12^^12^The Postal Museum (subsequently TPM) POST 30/1496, Discipline cases: particulars given on Form 141. Record ‘smelling of drink: not to be used’, 1907. Workers were not allowed to smoke on duty, and any hint of financial impropriety, such as involvement in betting or gambling, was also likely to lead to disciplinary action.^13^^13^See TPM POST 68/163, Rules and Instructions for Postmen in London, 1886; TPM POST 120/210, Questioning of suspected persons: methods pursued by officers of the Investigation Branch file XXI. Far worse, however, was the theft of mail which, once posted, had the legal status of the property of the Crown. As a correspondent to *St Martin’s-Le-Grand*, the Post Office’s staff magazine, stated:

No plea can excuse the thief, especially the letter stealer, whose express condition of service is a bond of honesty, sealed by oath made before entry upon those many duties which daily and hourly brings him in contact with public correspondence in the various post offices.^14^^14^‘An old time punishment’, *St Martin’s-le-Grand*, 17 (1907), 304.

That ‘bond of honesty’, however, was called into question whenever postal employees were suspected of interfering with the mail. From 1793 the Post Office employed a solicitor to investigate offences committed by its own employees, and this function grew in importance in parallel with the expanding volume of mail.^15^^15^TPM POST 30/1492, Confidential Enquiry Branch General Post Office (GPO): Revision, 1907. Historical summaries of Branch workings and grades employed, 1793–1907, folio V, 1; see also TPM POST 120/149 Committee on the Confidential Inquiry Branch: report and proceedings, 1900–1901. In 1848 an office known as the Missing Letter Branch was established specifically to investigate complaints about missing mail, reporting to the Inspector General in the central London headquarters, and in 1858 the complement of staff was strengthened with the addition of four travelling clerks and two Metropolitan Police constables seconded to the Post Office.^16^^16^TPM 340/1492, Confidential Enquiry Branch (GPO), folio V, 4. The clerks were expected to visit post offices where a problem had been reported and were given the responsibility of identifying dishonest workers, often through the use of ‘test letters’ which were posted with the deliberate aim of catching the culprit red-handed. In 1882, in light of the rising number of thefts by postal workers, the introduction of postal orders and the imminent inauguration of parcel deliveries, the Postmaster General and the Treasury sought to widen the remit of the Missing Letter Branch, and in the following year it was renamed the Confidential Enquiry Branch. The officer in charge was given the title of Director, indicating the growing importance of the role and also elevating the status of the travelling clerks.^17^^17^*Pall Mall Gazette*, 2 March 1882, 4. As well as a handful of staff at the central headquarters, the number of travelling clerks by then had risen to nine, with each clerk responsible for a particular region and based at one of the larger provincial offices. By 1893, the number of clerks had further increased to 16 operating across the entire country, assisted by the same number of policemen.^18^^18^See TPM POST 30/812 Investigation Branch (Formerly Confidential Enquiry Branch), Post Office Headquarters: revisions and general papers, 1890–1900, folio 15. There is remarkably little written on how the Post Office investigated internal theft of the mail. Standard histories rarely mention the Missing Letter Branch or its successors. Gavin McGuffie (Senior Archivist, The Postal Museum), personal communication, 15 February 2024.

**Figure 2.**
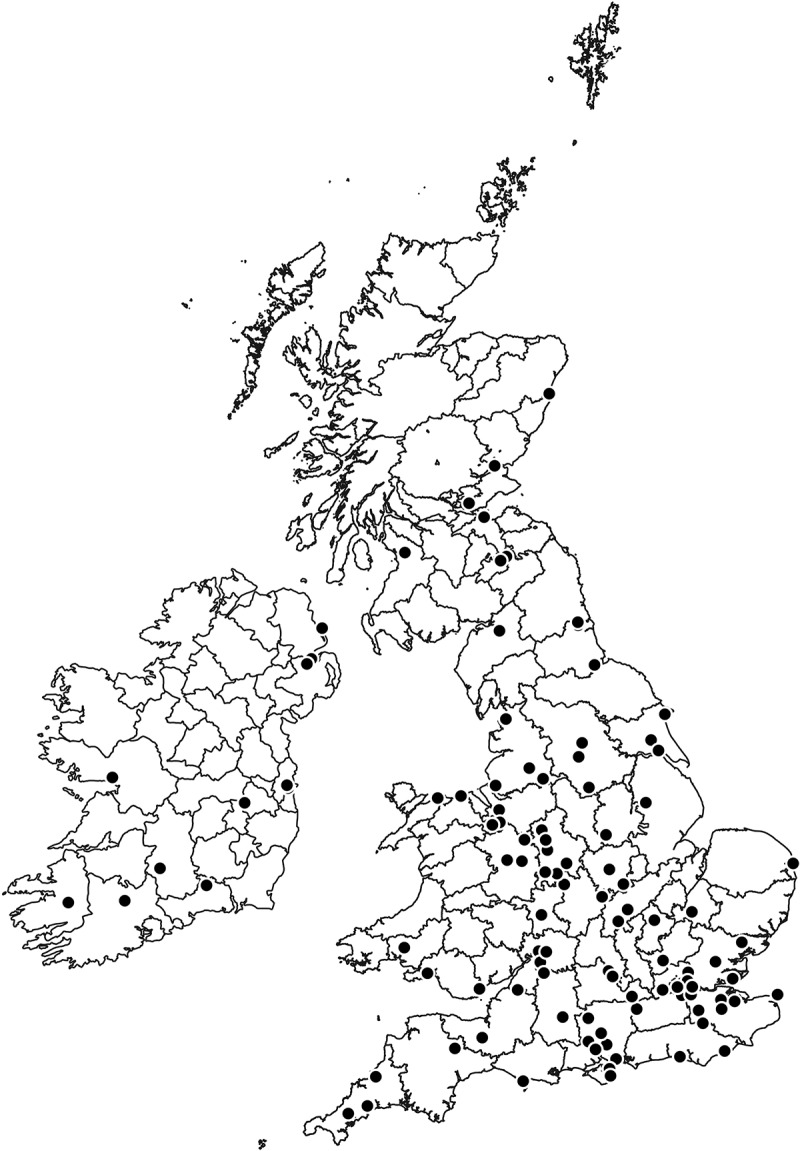
Weekly locations of the Confidential Enquiry Branch travelling clerks in the year ending 7 August 1895.

Source: TPM POST 120/42, weekly circulars issued to the travelling clerks by the Confidential Enquiry Branch regarding the movements of the investigation officers during the week.

Whenever a number of complaints were received from the public about missing mail, investigations were initiated and a clerk from the Confidential Enquiry Branch together with a police constable seconded to the Post Office would then be despatched to the post office in question. In some cases, the efforts made to visit even the most remote post offices hinted at the importance of the task. In July 1894, for example, Mr Madde, one of the travelling clerks responsible for Ireland, reported back on his travels in Galway Bay, where after a 20-mile journey by horse he took a coracle to visit an island two miles offshore, followed by a five-mile walk to where his investigations took place, returning the following day. In subsequent months he visited Dublin, Larne, Belfast, Donnybrook, Mallow, Killarney, Cork and Lisburn, before travelling back to England and there visiting London, Wokingham, Manchester and Liverpool.^19^^19^TPM POST 120/42, Weekly circulars issued to the travelling clerks by the Confidential Inquiry Branch regarding the movements of the investigation officers during the week. Other clerks similarly reported their weekly movements back to central headquarters, explaining where they had been and the cases they had investigated. Using information about where each clerk was at the end of the week, we can reconstruct the geographical scope of their activities, as shown in Figure 2 for a 12-month period ending in August 1895.^20^^20^BPP, 1884, LII, Estimates for Revenue Departments, 1884–85, 183–84; BPP, 1895, LXVI, Estimates for Revenue Departments 1895–96, 54. The locations shown on the map were those reported at the end of each week. The map highlights 116 places from which the travelling clerks reported at the end of each week, with many other places being visited in the intervening days, and demonstrates clearly the national scope of the Confidential Enquiry Branch investigations, including both large urban centres and more remote rural parts of the country.

The Post Office went to some pains to determine the guilt of employees suspected of theft. In some cases a clerk posing as a new hand who was unknown to the workforce would work alongside the suspect to observe his actions. Enquiries would also be made about the company kept by a suspect or whether he was involved with gambling or drinking.^21^^21^‘Experiences of a Post-Office detective’, *The New Zealand Graphic and Ladies Journal*, W. Coen, 17 February 1892, 154. The usual practice, however, was for the travelling clerk to send a test letter containing marked coins or stamps, or other valuable items, which would then be tracked as it moved through the different stages from sorting to delivery.^22^^22^Austin Phillips, ‘Crime in the British Post Office’, *Strand Magazine*, 34 (1907), 409–10. If the letter failed to be delivered intact to the intended recipient, the worker under suspicion would be challenged and searched.^23^^23^See, for example, *The Bury and Norwich Post*, and *Suffolk Herald*, 27 November 1860; *Northern Echo*, 10 October 1891, 4. This often happened in places such as the toilets, where an employee could open a letter unobserved by their colleagues. During the 1860s a plumber had been specifically employed in London to make sure that toilets in the various district offices were watched for this kind of activity, but after his death in 1866 Frederic Hill, the brother of Sir Rowland Hill, founder of the penny post and later Secretary to the Post Office, recorded that thefts of mail had increased ‘by the discontinuance of a check to prevent the water closets being used as places to get rid of inculpating evidence’.^24^^24^TPM POST 120/499, Historical and personal accounts of the Investigation Branch, cases and working conditions. The role was subsequently reinstated in 1873, and by 1907 at least five men were employed as ‘watchers’ specifically to keep an eye out on workers suspected of theft.

**Figure 3.**
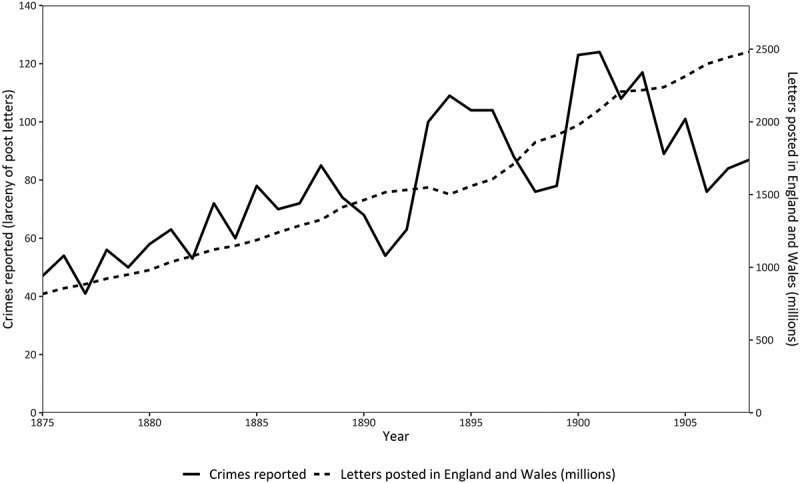
Crimes reported for theft of the mail in England and Wales, 1875 to 1908.

Source: Indictments are derived from figures in British Parliamentary Papers (BPP), Return of Judicial Statistics of England and Wales (Part I. Police; Criminal Proceedings; Prisons), 1875 to 1908; the number of letters delivered are derived from the Postmaster General’s annual reports, 1875 to 1908.

The extent of theft can be gauged with reference to the number of Post Office workers indicted for stealing the mail in England and Wales between 1875 and 1908, shown in Figure 3. The figures refer to the offence of larceny by servants of the Post Office, which was treated as a distinct indictable crime tried at a higher court. In England and Wales over the period, there were nearly 2700 postal workers indicted for mail theft, almost all of whom were found guilty. The general upward trend of indictments, albeit punctuated by falls in the late 1880s, the mid-1890s, and again in the early 1900s, corresponds closely with the growing volume of mail sent through the post, though other factors also played a part. The introduction of postal orders in 1881 as a means of transmitting small sums of money relatively cheaply was thought to have provided more opportunities to steal from the Post Office. Their popularity was enormous and immediate: within 10 years of their introduction over 778 million postal orders had been issued by the Post Office, with their use extending not just to the United Kingdom but throughout the empire.^25^^25^*Liverpool Mercury*, 3 January 1900, 4. The amount of money transmitted by the new postal order arrangements rose in parallel to their popularity: in 1882–1883 over £3.4 million was transmitted, rising to over £19 million by 1890–1891 and to over £30 million by 1900–1901.^26^^26^Daunton, *op. cit*., 92. Mail order business boomed following the introduction of postal orders, and newspapers were filled with adverts offering products in return for payment by postal order. At first, however, the poor design and the careless completion of postal orders by the public made it relatively easy to forge recipients’ signatures.^27^^27^See, for example, *Illustrated Police News*, 24 December 1881, 3; *Jackson’s Oxford Journal*, 1 February 1890, 8; *York Herald*, 20 December 1890, 5; *Blackburn Standard*, 29 August 1891, 6; *Huddersfield Chronicle*, 7 April 1892, 3. As the *Birmingham Daily Mail* noted in 1885, in an article entitled ‘Dishonesty of the Post Office’, ‘“Postal order thieves”, as they are termed, are now a recognised class of criminals’.^28^^28^*Birmingham Daily Mail*, 29 May 1885, 3. In subsequent years, new designs and improved arrangements for payment made it more difficult to fraudulently redeem postal orders, but suspicion remained that they were responsible for an apparent upsurge in theft by postal workers.^29^^29^Hansard, 4th series, 38 (1896), 24–25. The Post Office authorities, however, were understandably keen to maintain public confidence in the system, and disputed whether the increased use of postal orders was the reason for any rise in convictions, citing instead the practice of employing more auxiliary postmen who they thought were less honest than full-time ‘established’ workers.^30^^30^See BPP, 1897, XLIV, Inter-Departmental Committee on Post Office Establishments: Minutes of Evidence, Indices, Summaries, Appendices, evidence of Lewin Hill, q. 11936.

In most cases, workers prosecuted for mail theft were found guilty, a fact attributed by the Post Office authorities to the use of test letters and the care taken to ascertain guilt ‘with absolute certainty’ before attempting to prosecute.^31^^31^BPP, 1896, XLIV, Return of Judicial Statistics of England and Wales, 1894 (Part 1), 27. If found guilty, workers faced not only a lengthy prison sentence but also dismissal and loss of any pension rights they might have accrued. Under the terms of the Post Office (Offences) Act of 1837, workers guilty of interfering with or stealing from the mail could be transported, although this punishment ceased to be used after 1856 and was replaced by imprisonment.^32^^32^Post Office (Offences) Act 1837 1 Vict. c.36.The last person to be sentenced to transportation at the Old Bailey was Cornelius William John Hardy in 1856. See *Old Bailey Proceedings Online* (www.oldbaileyonline.org, version 8.0, 14 July 2023), 3 March 1856, trial of Cornelius William John Hardy (t18560303-347). In the 1860s it was not uncommon for those found guilty at the Old Bailey, for example, to be sentenced to terms of up to six years, depending on the severity of the offence and the character of the individual in question. Where the theft of mail involved postage stamps or money orders, guilty verdicts normally carried a sentence of up to 18 months, but this was often raised to five or even six years when valuable items had been stolen, such as jewellery or gold sovereigns. In 1875, for example, Henry Woodgate was sentenced to five years’ penal servitude at the Old Bailey for stealing letters containing two £5 bank notes, but on the same day an even harsher sentence of six years was handed to Lewis Ovenden, a 37-year-old sorter at the main headquarters in London, ‘who was in a position in the Post Office of greater trust and confidence’ and who was therefore dealt with more harshly by the courts than was the usual practice.^33^^33^*John Bull*, 26 September 1874, 658; *Old Bailey Proceedings Online*, 21 September 1874, trial of Henry Woodgate (t18740921-371); Old Bailey Proceedings Online, 21 September 1874, trial of Lewis Ovenden (t18740921-375). In 1886 an even longer sentence of 10 years’ penal servitude was bestowed on George Greenland, a 49-year-old postman who had worked at the General Post Office for upwards of 20 years. Over a period of time he was said to have stolen property to the value of £1300, including a cheque book, a money order worth £35, and a postal order for £34, in addition to forging receipts for several postal orders.^34^^34^*Old Bailey Proceedings Online*, 11 January 1886, trial of George Greenland (t18860111-170). *Middlesex County Times*, 16 January 1886, 2. Although his sentence was exceptional, he was not the only one to be dealt with harshly. Two years later, William Chart was apprehended by a clerk from the Confidential Enquiry Branch as he attempted to cash three forged postal orders, and when his lodgings were searched a mail bag containing upwards of £1000 together with about 300 letters and other valuables was found. He too received a sentence of 10 years’ penal servitude.^35^^35^*Old Bailey Proceedings Online*, 28 May 1888, trial of William Chart (21) and Edward James Smith (21) (t18880528-517); *Cambridge Independent Press*, 4 May 1888, 3; *Newcastle Chronicle*, 2 June 1888, 2. These cases involved exceptionally large amounts stolen in a systematic way over a relatively long period of time, but most thefts of mail were of smaller value and sentences were accordingly lower. Between 1875 and 1884, postal workers found guilty of mail theft at the Old Bailey on average received jail sentences of around three and a half years, though this term fell in the remaining years up to 1900 to around 19 months.^36^^36^We are very grateful to Saskia Alais, who provided this information based on an analysis of 564 cases of theft of mail tried at the Old Bailey between 1875 and 1900. The original data is derived from Tim Hitchcock, Robert Shoemaker, Clive Emsley, Sharon Howard and Jamie McLaughlin, *Old Bailey Proceedings Online, 1674–1913* (www.oldbaileyonline.org, version 7.0, 24 March 2012).

The desire to prosecute workers was matched by the frequency with which trials for mail theft were reported in the newspapers, though at times doubt was cast as to whether it was in the interest of the Post Office to make public the true extent of crimes.^37^^37^*Leeds Mercury*, 7 September 1861, 6. When reports of trials appeared in the press, they often noted the name of the travelling clerk before describing the efforts made to confirm exactly who was involved and how the crime took place.^38^^38^See for example, *Morning Post*, 30 April 1888, 3; *Western Morning News*, 25 June 1888, 5; *Berrow’s Worcester Journal*, 6 April 1889, 2; *Birmingham Daily Post*, 2 September 1889, 5; *Manchester Weekly Times*, 7 September 1889, 3; *Faversham News and East Kent Journal*, 12 September 1891; *Canterbury Journal and Farmer’s Gazette*, 12 December 1891; *The Standard*, 7 April 1892, 6. Cases could excite considerable local interest, particularly when there had been numerous thefts of mail. In 1885, for example, Mary Derry, the daughter of the local postmaster at Burslem, was tried following an investigation by Robert Bradford of the Confidential Enquiry Branch which involved a test letter that included two marked gold sovereigns. Perhaps because of its unusual nature – it was rare for a woman and a member of a postmaster’s family to be accused of theft – the case attracted considerable interest and the approaches to the town hall, where the case was being heard, were thronged with several hundred curious spectators.^39^^39^*Birmingham Daily Post*, 21 January 1885, 8. The jury returned a guilty verdict but with a recommendation of mercy and Mary received a relatively light sentence of eight months’ hard labour.^40^^40^*Newcastle Guardian*, 21 February 1885, 6. The interest shown in Mary Derry’s case, however, was far from unique, and other cases, such as that of William Hinton, a letter carrier from Kidderminster, were also widely reported in the local press.^41^^41^*Berrow’s Worcester Journal*, 30 January 1886, 7; *Birmingham Mail*, 19 May 1886, 3. See also the case of John Proudfoot, *Ross-shire Journal*, 5 May 1882. Hinton was accused of stealing two half sovereigns and 60 stamps, but when he was confronted by the travelling clerk sent to investigate the theft he was also found with a betting book and this sealed his fate. Postal workers were expressly forbidden to gamble or bet on pain of dismissal, and perhaps for that reason Hinton received a lengthy sentence of five years with hard labour.^42^^42^*Nuneaton Advertiser*, 6 February 1886. See POST 68/163 Rules and Instructions for Postmen in London, 1886.

The Post Office authorities were keen to prosecute offenders not merely as a deterrent to other workers but also to reassure the public that the mail which had been entrusted to them was secure. This related not just to letters or parcels containing valuable items but also to confidential messages sent through the post. Indeed, assuring the public of the sanctity of the mail touched on contested issues of government surveillance, a topic that was of particular concern in the 1870s and 1880s following a set of embarrassing leaks by poorly paid copyists in the Civil Service.^43^^43^See J. Purdon, ‘Secret agent, official secrets: Joseph Conrad and the security of the mail’, *Review of English Studies*, 65 (2013), 305, 312. For further discussion see D. Vincent, *The Culture of Secrecy: Britain 1832–1998* (Oxford, 1999).

It was a delicate balance, however, between reassuring the public that the majority of postal workers were reliable and honest while at the same time seeking to bring the full weight of the law to bear on those workers who transgressed the rules. In 1896 the Postmaster General claimed that on average there was a high level of honesty within the workforce, noting, ‘That the public repose full confidence in the Post Office is clearly established by the constant increase in the Money and Postal Orders and other valuable articles sent by post’, adding a warning to the public, however, that valuables or money orders should always be registered, ‘not only in their own interest, but also in the interest of my staff’.^44^^44^Postmaster General’s 42nd Annual Report (1896), 21. In response to a Parliamentary question about the number of convictions for larceny, he reported that he was ‘glad to think that the proportion of the staff guilty of dishonesty is very small’, equivalent to roughly one conviction for every 2500 workers employed, though a Parliamentary return in the previous year suggested that the figures were closer to around one in every 1400 workers.^45^^45^Hansard, Fourth Series, vol 38 (1896), 1150–1151; BPP, 1895, LXXX.315, Return of Number of Postal and Telegraph Employees; Number charged with Theft or other Crime, 354.

In addition to the need not to alarm the public unnecessarily by focusing on thefts, there were also issues raised about careless postage habits and the use of test letters. Sending valuable items by post without registering them, for example, or failing to include details of the payee and the relevant post office on postal orders – making it much easier for others to cash them – were seen as inducements to commit theft, particularly by poorly paid workers.^46^^46^*Birmingham Daily Post*, 2 September 1889, 5. Test letters also raised similar concerns, and at times the Post Office itself could find itself on trial, along with the dishonest workers that it sought to prosecute. When Margaret Eagelson, a charwoman in the Post Office, was accused of stealing a test letter containing five shillings, the judge blamed the Post Office authorities for leaving the letter in a prominent position on a desk, knowing that she would clean there. Although found guilty, Justice Williams sentenced Eagelson to a mere day in prison, which had already been served, and she was therefore discharged.^47^^47^*Lancaster Gazette*, 5 December 1891. The main complaint, however, about the use of test letters was voiced by Charles Durrant, a sorter in London and representative from the Fawcett Association, the postal clerks’ trade union, in his evidence to the Parliamentary Committee on Post Office Wages in 1904. Durrant argued that the main reason for theft was low wages and that test letters were merely forms of entrapment for poorly paid workers: ‘To pay a man an insufficient wage and deliberately offer him facilities to steal is immoral and un-English’.^48^^48^BPP, 1904, XXXIII, Committee on Post Office Wages. Minutes of evidence, q. 269. The evidence provided to the Parliamentary Committee by members of the Fawcett Association stated that 24 shillings a week was the lowest possible amount – the ‘moral minimum’ as they put it – upon which a sorter should be asked to live until he received his first increment, and yet many workers earned well below this amount.^49^^49^*ibid*. In 1899, when postal worker Edward John Larkman was convicted at the Old Bailey of stealing two postal orders, for which he received a sentence of 18 months’ hard labour, his earnings amounted to less than 21 shillings a week. This was typical for employees of his grade, and comparable to Charles Booth’s minimum required for a moderate-sized family to remain above the poverty line.^50^^50^*Old Bailey Proceedings Online*, 9 January 1899, trial of Edward John Larkman (21) (t18990109-107). For Booth’s poverty line see A. Gillie, ‘The origin of the poverty line’, *Economic History Review*, 46 (1996), 715–30.

Such considerations spoke more widely to public concerns about the Post Office’s labour practices. While prosecutions were seen as an important deterrent to untrustworthy workers, there was also the possibility that the integrity of the Post Office itself could be questioned, as Durrant and other trade unionists had suggested. The employment of young boys in responsible positions as telegraph messengers, for example, was seen as a risky practice that placed immature and poorly paid workers in the path of temptation.^51^^51^*Globe*, 16 December 1879, 2. In 1875 a 14-year-old sorter, Henry Busby, was given a four-week sentence for stealing and then destroying a letter – a relatively lenient judgement on account of his youth and the small amount of pay he received.^52^^52^*Old Bailey Proceedings Online*, 1 March 1875, trial of Henry Busby (14) (t18750301-198). Two years later another young sorter, Vincent Taverner, also received a lenient sentence for a similar offence, also on account of his youth and the temptation to which he had been subjected.^53^^53^*Old Bailey Proceedings Online*, 19 November 1877, trial of Vincent Augustus William John Taverner (15) (t18771119-22). Such misdemeanours, however, were not isolated events and other scandals during the 1870s relating to the sexual (mis)conduct of telegraph boys in London raised questions about the morality of the Post Office’s youthful employees.^54^^54^See K. Hindmarch-Watson, ‘Male prostitution and the London GPO: telegraph boys’ “immorality” from nationalisation to the Cleveland Street Scandal’, *Journal of British Studies*, 51 (2012), 594–617. The discovery of illicit homosexual activities involving telegraph boys in a male brothel in Cleveland Street in 1889 came about during an investigation of mail theft and served to underline the continued failure of the Post Office to weed out vice among its younger workers who were exposed daily not just to financial temptation but also to the dangers of moral corruption.^55^^55^K. Hindmarch-Watson, ‘Reframing the Cleveland Street Scandal in England: telegraph boys, the service economy, and compensated sex’ in J.G. Scott, C. Grove and V. Minichiello (eds), *The Routledge Handbook of Male Sex Work, Culture and Society* (Abingdon, 2021), 12–37.

Despite the fact that most workers brought before the courts were found guilty, judges were not necessarily sympathetic to the way in which the Post Office investigated suspected cases of theft or misdemeanour. In 1892 Judge Vaughan Williams, a well-known London lawyer and judge adjudicating the case of a postal worker accused of theft, questioned whether the practice of sending a clerk from the Confidential Enquiry Branch – ‘a skilled and irresponsible cross-examiner, enthusiastic for evidence on which to prosecute his man’ – was an appropriate way to detect guilty workers.^56^^56^TPM POST 120/23, Comments and criticisms of investigation methods, folio 50. He was reported as stating that ‘If the Post Office wanted to secure the conviction of guilty servants, the right way was to treat their servants at large like honest men’.^57^^57^*ibid*., folio 51. In response the Post Office authorities defended their right to conduct a verbal interrogation, arguing that this also provided an opportunity for the worker accused of theft to explain his actions and prove his innocence.^58^^58^*ibid*., folio 51–54. By the early twentieth century, some observers were beginning to blame mail theft on the heavy duties carried out by postmen and sorters, as well as their insanitary workplaces and meagre wages.^59^^59^H.L. Adam, *The Story of Crime: From the cradle to the grave* (London, 1908), 259. In the case of Arthur Pye, a 39-year-old sorter found guilty in 1907 of stealing mail, a lenient sentence of three months was passed when the Recorder stated that given the conditions in the Post Office it was ‘impossible (for the defendant) to act as he might have done otherwise’.^60^^60^*Old Bailey Proceedings Online*, 10 December 1907, trial of Arthur Pye (39, porter) (t19071210-5). Although Pye had been on trial and had broken the trust vested in him as a public servant, it was almost as if the Post Office itself was the main culprit behind the crime. Prosecuting workers who transgressed the rules, therefore, involved the Post Office in a delicate balance between reassuring its customers that the institution could be trusted while at the same time not alienating a public sympathetic to poorly paid servants of the state – a balance that sometimes the authorities failed to achieve.

## Sickness, pensions and the problems of malingering

The complex nature of trust identified in the way in which theft was addressed was also evident in how sickness was dealt with in the Post Office. Like other branches of the Civil Service, permanent employees – known as being on the ‘establishment’ – were entitled to sickness pay and, once they had worked for at least 10 years, became eligible for a pension. In both cases, the Treasury bore the cost which was counted against the revenue raised through the Post Office’s operations. The provision of sick pay was important for several reasons: it helped to ensure that employees who were unwell did not spread infection to the rest of the workforce, or indeed infect the mail itself, and it also meant that sick workers forced to take time off did not suffer financial hardship which might then have encouraged them to steal from the mail.^61^^61^For the way in which sick pay was provided see K. McIlvenna, D. Brown and D.R. Green, ‘“The natural foundation of perfect efficiency”: medical services and the Victorian Poor Law’, *Social History of Medicine*, 33 (2020), 539–58. From the 1870s the normal practice was to provide full pay for up to six months of sick leave and then half pay for a further six months, providing that there was a reasonable chance of the worker returning to their job. In some situations, even this could be extended at the discretion of the authorities in London. At the same time, however, it was important to control the financial burden of sick leave. To achieve this the Post Office set up a comprehensive medical service that employed doctors on a permanent and part-time basis to examine candidates to ensure that they were fit for service, and to monitor sickness claims once they were taken onto the establishment.

The importance of these checks on workers’ health became increasingly evident over time as the amount of sick leave taken by Post Office workers began to increase.^62^^62^For discussions relating to sick leave see D. Brown, D.R. Green, K. McIlvenna and N. Shelton, ‘The beating heart of the system: the health of postal workers in Victorian London’, *Journal of Historical Geography*, 68 (2020), 75–85; D.R. Green, D. Brown and K. McIlvenna, ‘Addressing ill health: sickness and retirement in the Victorian Post Office’, *Social History of Medicine*, 33 (2020), 559–85; D.R. Green, D. Brown and K. McIlvenna, ‘“The postman wears out fast”: retiring sick in London’s Victorian Post Office’, *London Journal*, 44 (2019), 180–205. Using information about sick leave derived from the analysis of 26,500 pension records of workers who retired from the Post Office between 1860 and 1908, it is possible to construct a time series of sick leave for the period. The pension records include for most workers the amount of sick leave taken in the 10 years prior to retirement, and we can use this information to reconstruct the pattern of sickness. In order to limit the possibility that the figures could be skewed by workers who were forced to retire because of poor health, the analysis here only uses information for the first eight years of that period. In effect, this means that we only use information for workers more than two years prior to retirement. With this information we can construct three complementary measures that together identify the nature and extent of sickness in the Post Office: the average number of sick days per worker; the proportion of workers who took sick leave in any given year; and the total amount of working time lost as a result of sickness. The reason why these measures are important is because they represent a real and growing financial burden which the state had to bear, and for that reason it was important to be sure that the claims for sick leave were made for valid reasons.

**Figure 4.**
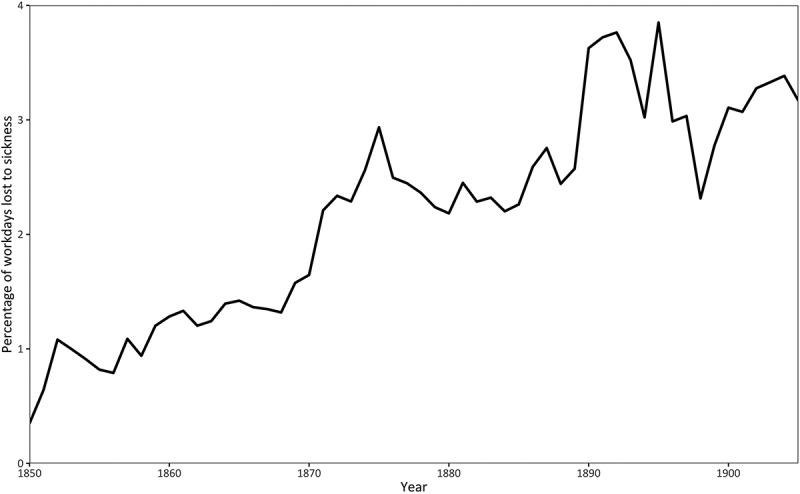
Number of days lost to sickness, 1850–1908.

Source: TPM POST 1, Treasury Letters, 1860 to 1908; accessed via the Addressing Health database at www.addressinghealth.org.uk. The data are derived from workers who retired between 1860 and 1908, but contain information on their sickness absences from 1850 onwards.

**Figure 5.**
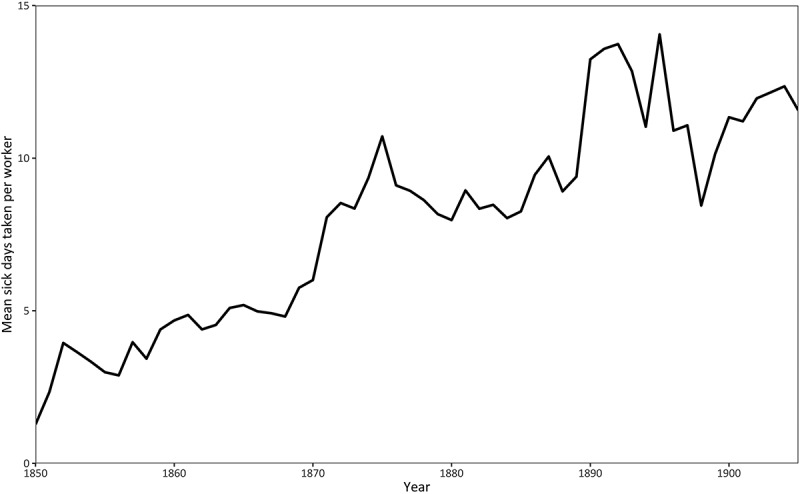
Average number of days off sick, 1850–1980.

Source: TPM POST 1, Treasury Letters, 1860 to 1908; accessed via the Addressing Health database at www.addressinghealth.org.uk. The data are derived from workers who retired between 1860 and 1908, but contain informa tion on their sickness absences from 1850 onwards.

**Figure 6.**
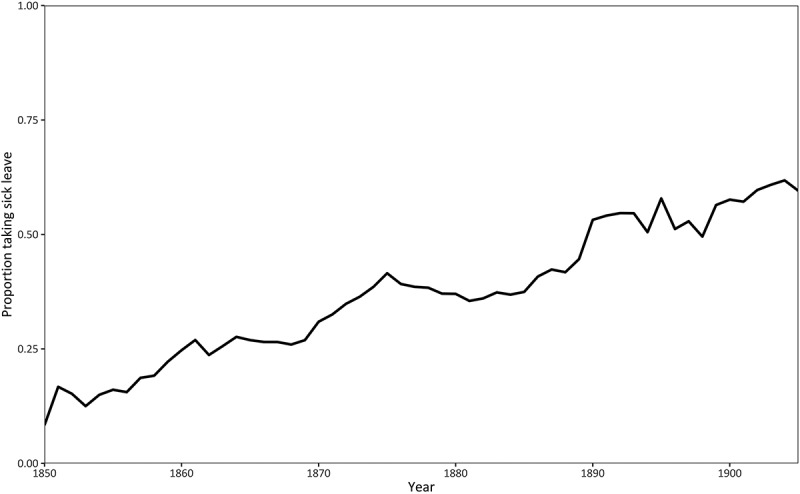
Proportion of workers taking sick leave, 1850–1908.

Source: TPM POST 1, Treasury Letters, 1860 to 1908; accessed via the Addressing Health database at www.addressinghealth.org.uk. The data are derived from workers who retired between 1860 and 1908, but contain informa tion on their sickness absences from 1850 onwards.

The proportion of days lost to sickness, shown in Figure 4, attempts to assess the impact of sickness on the Post Office’s work as a whole. It is based on the total number of sick days each year identified in the pension records divided by the number of total working days, assuming that the latter measure was based on an annual figure of 365 days. It shows that over time, the relative importance of sickness absence increased and that by the end of the nineteenth century, at least 3% of working time was being lost as a result of poor health. There could be two possible explanations for this trend: the average amount of sick leave per worker could be increasing and the proportion of workers who took sick leave could similarly have been rising. In fact, both situations were true. Figure 5 shows that the average amount of sick leave taken each year rose and by the end of the period stood at around 12 to 13 days. Figure 6 shows that over the same period, a greater proportion of workers took sick leave in any given year. Therefore, it was not just the length of sick leave that was rising, but also the proportion of the workforce claiming sick leave. Both these trends were responsible for a growing problem of having to finance increasing amounts of sick pay.

The emerging problem of sickness identified above had clear financial implications for the profitability of the Post Office. To counter the risk of spurious claims, the Post Office created what can best be described as a sanitary bureaucracy – a complete administrative system of standardised forms, recording and reporting – to monitor sickness rates: doctors were employed to check on sick workers in their own homes as much to prove that absences were genuine as to provide care; sick absences were carefully noted as part of a worker’s employment record and, from the 1890s, compared to similar workers at the same post office in order to assess whether or not they remained capable of fulfilling their duties effectively.^63^^63^This concept is borrowed from T. Crook, *Governing Systems: Modernity and the making of public health in England 1830–1910* (Oakland, 2016), 106–20. Employees who were found to be taking abnormally long sickness leave were under threat of being pensioned off, while those thought to be malingering were disciplined.

The process of monitoring sickness fell to the Post Office Medical Service, established in 1855. A handful of doctors were employed on a full-time basis at the central headquarters in London and in a few other large cities, and many more part-time doctors elsewhere were paid on a per capita basis.^64^^64^For further discussion of the Post Office Medical Service see McIlvenna et al., *‘*The natural foundation of perfect efficiency’, *op. cit*. The service expanded rapidly, especially after the Post Office took over the inland telegraph companies in 1870, which brought many more female employees into the workforce, and as the workforce continued to grow, so too did the number of doctors.^65^^65^K. Hindmarch-Watson, *Serving a Wired World: London’s telecommunications workers and the making of an information capital* (Oakland, 2020), 16. In July 1886 Sir Arthur Blackwood, Secretary to the Post Office, wrote to the Postmaster General in relation to a request to extend the medical service, stating that ‘It is impossible to exaggerate the importance of this system of Medical Supervision as a means of checking absence on a false or insufficient plea of illness and of arresting illness in its incipient stages’.^66^^66^TPM POST 64/1, *Post Office Medical Service*, 304. By 1900 the Post Office Medical Service employed nearly 600 doctors, including five female doctors to care for the growing number of women in the workforce.^67^^67^Figures are taken from the Post Office Chief Medical Officer’s annual reports which began to be published from 1891. See TPM POST 64/16, ‘Chief Medical Officer’s annual report and tables of sick absence’ 1893–1900; POST 64/27, ‘Chief Medical Officer’s annual report and tables of sick absence’ 1891; POST 64/28, ‘Chief Medical Officer’s annual report and tables of sick absence’ 1892.

Post Office doctors had several responsibilities, that started with the physical examination of candidates to ensure that they were fit for duty and thereafter involved certifying sickness absences and providing care for workers. However, one of their primary responsibilities was ‘to inquire into cases in which there is suspicion of feigned or exaggerated illness’.^68^^68^H. Bashford, ‘The Post Office Medical Service’, *Post Office Green Paper 31* (London, 1936), 3. The reasons for such suspicion could be very slight and, arguably, obscure but it was the duty of each Post Office doctor to satisfy themselves that the reasons for any sick absence were genuine. Given the rising sickness levels identified above, this responsibility was of increasing importance to the efficient and economical running of the Post Office.

Having been certified unwell and unable to work, it was then the responsibility of the sick employee to behave in an appropriate manner. In 1876 Mr Wells, a clerk based in Hull, was requested by the Medical Officer not to venture out in the evenings on account of his poor health.^69^^69^TPM POST 64/1, *Post Office Medical Service*, 898. However, it was soon noted that on several occasions Wells had been seen visiting the theatre after dark, which the Post Office doctor suggested was the cause for the severe cold that added a further five days of sickness. Under those circumstances, his superior officer ‘recommended that he should be allowed no sick pay for the period in question and that he should be reduced by two places on his class’.^70^^70^*ibid*., 898–99. Although it was stressed that Mr Wells was not suspected of malingering, he was at the very least ‘guilty of imprudence in being out of doors when he had better have been in’. The Post Office doctor’s opinion in this case was crucial in determining the outcome arising from the wilful disobedience of instructions, and for that irresponsible behaviour Wells was docked wages and refused sick pay.^71^^71^*ibid*., 899.

In practice, the regulations requiring the certification of sickness were complex and varied between different occupational groups, grades of workers, and between different places, raising questions about trust and equity not just on the part of the worker but also on the part of the certifying doctor. A worker who was sick either had to visit the local Post Office doctor or, if too unwell to attend, to send a note to inform a superior officer. Provided they lived within a specified distance of their normal place of work, usually three miles, they would then be visited by the local Post Office doctor. If the local office was too small to warrant the appointment of a doctor, then the worker had to use another doctor to certify sickness but at their own expense, and this had to be ratified by the Post Office authorities.

The use of private doctors brought issues of trust to the fore. In London, pleas of illness were not accepted as an ‘excuse for absence for a single day unless supported by a Medical Certificate either from the Medical Officer or some qualified Practitioner’.^72^^72^*ibid*., 207. Since many London workers lived several miles away from their normal place of work, it was relatively common for sick workers there to visit their own doctor. However, if a medical certificate was provided by a practitioner not connected with the Post Office, it was sent to a Post Office medical officer to be examined and then countersigned.^73^^73^TPM POST 64/10, ‘Manual for the use of Post Office Medical Officers’ (London, 1913), 8–9. There were strict regulations regarding the information that these certificates needed to provide, including the nature of the complaint and the likely length of absence.^74^^74^TPM POST 64/1, *Post Office Medical Service*, 939. In addition, they had to be dated, and contain the address and name of the medical practitioner who had provided the certificate. Only then could the worker be considered eligible to receive sick pay.

The use of private doctors to certify sickness, however, raised concerns from the Post Office authorities, not least because they thought that lenient doctors would be sought out by workers keen to be signed off sick or who wished to conceal the nature of their illness.^75^^75^*ibid*., 366–69. There was a view in the Post Office that private medical certificates ‘were very commonly obtained where the absentee was suffering from some complaint the nature of which he wished to keep the Departmental Medical Officer in ignorance of’, perhaps ‘intemperance or some other cause within the control of the absentee’.^76^^76^*ibid*., 511. However, a more common concern was that private doctors were likely to be more lenient when it came to certifying the reasons for sickness absence. ‘There can be no doubt’, one observer noted in 1876, ‘that the Certificates we receive in excuse for non-attendance except when given by our own Medical Officers are not always to be implicitly relied on’.^77^^77^*ibid*., 226. One postmaster stated that ‘I believe that in nine cases out of ten the Medical Certificates furnished [by medical officers who do not belong to the department] were obtained under false pretences’.^78^^78^*ibid*., 227.

Where workers were thought to be dishonest, the opinion from a private doctor was treated with even more suspicion. In 1901, following an accident where he had supposedly fallen over a trolley and grazed his leg, Mr Nay, a postman in London, went to a private doctor to obtain a certificate to confirm that he was unable to work.^79^^79^*ibid*., 645–46. When asked why he had gone to a private practitioner, Nay stated that he had felt very unwell and so thought it advisable to seek the nearest doctor. Yet, the district postmaster noted that Nay bore ‘an indifferent character’ and was not to be trusted. He was ‘a very shifty man’ and the postmaster suggested that no private medical certificates offered by Nay should be accepted.^80^^80^*ibid*., 646. Evidently, the skills of private practitioners at detecting malingerers were not always felt to be up to the Post Office’s standards, and this problem was accentuated when it came to untrustworthy employees.

Despite the fact that written rules existed regarding certification of sick leave, implementing those in a uniform way proved more difficult. Enquiries by the Post Office found that policy and practice varied widely, with some places requiring immediate notification of sickness while in others two or even three days’ absence was the norm.^81^^81^TPM POST 31/17A, Sick leave one day absences 1878–1902, folio 1, 5. The notification period could differ depending on a worker’s grade, and here the issue of trust was bound together with status. For higher-grade workers, such as postmasters and supervisors, it was the practice for sickness absence only to require certification after three days. To require a medical officer to visit after one day’s absence, it was suggested, ‘would imply a suspicion that the cause assigned for absence is questioned’.^82^^82^See TPM POST 31/17A, Sick leave one day absences 1878–1901. For lower grades of clerical workers, it was normal to request certification from the second day of absence.^83^^83^TPM POST 31/17A, Sick leave one day absences 1878–1901, ‘Memorandum to the staff employed at the Central Telegraph Station and Stock Exchange Office’, January 1873.

Practice also differed depending on place. In London, the rules for countermen and telegraphists at the Central Office stated that a medical certificate had to be furnished on the first day of absence through illness, though it was also noted that this requirement had not been enforced strictly.^84^^84^*ibid*., folio 3. In Dublin, the practice of allowing two days’ absence without certification had first been applied to female staff but, despite reservations, it then spread to male employees. In 1885 Dr Fitzgibbon, the Dublin Post Office doctor, warned against the practice for male officers, arguing that ‘The effect of it is to encourage habits of intemperance, dissipation and malingering’, adding that the list of names of absentees ‘always contains those of men known to be of irregular habits’.^85^^85^*ibid*., folio 2. In the smaller post offices, where no doctor was appointed, it was felt harsh to compel workers to furnish a certificate at their own expense for a single day’s absence, and therefore they only had to do so from the second day, though all sick days were still to be noted on the worker’s record.^86^^86^TPM POST 31/17A, Sick leave one day absences 1878–1901 file number XIII: Question of requiring medical certificate for one day’s absence. However, in 1891 it was found that in some urban areas a medical certificate only had to be provided on the second day of a period of sickness absence. An enquiry launched to discover how practices differed across offices found that in Liverpool a certificate was still required after only one day of illness but that in Birmingham and Manchester certificates were not required for one-day absences except when malingering was suspected. In Belfast, clerks complained that they were ‘refused the privilege of sick leave for one day without the necessity of providing a certificate’, and they appealed to the Postmaster General, drawing attention to their English counterparts who only had to report sickness on the second or sometimes the third day of absence: ‘The trust reposed in the honour of English Clerks has not been abused. Will it be too much to ask that faith be exercised to the same extent in Irish Clerks?’^87^^87^TPM POST 64/1, *Post Office Medical Service*, 374–75.

In practice, there was no general rule regarding the certification of sickness but rather a series of decisions made on a case-by-case basis, dependent on grade and geography, and under these circumstances workers could argue a case for discretion and trust. As is apparent from the appeal from the Belfast clerks, the requirement to provide a medical certificate for short absences was seen by some as an attack on workers’ integrity. In this instance, their appeal was answered and it was decided that ‘the English practice should be followed’, but the matter of trust between workers and the Post Office authorities remained an issue.^88^^88^*ibid*., 378. In 1881, a protest was submitted by two telegraph superintendents in Liverpool. Given their position, the pair objected to having to provide a medical certificate before the third day of absence. The Secretary responded that there was ‘no ground whatever’ for a different practice and that they had to ‘conform to the rule’ and ‘readily admit that a special exemption in their favour is out of the question’.^89^^89^*ibid*., 254. Others described these protesting employees as ‘unreasonable’, and also pointed out that ‘it is not as though something were being enforced upon them from which others were exempt’.^90^^90^*ibid*., 255. While uniformity of treatment might have been the desired outcome from central headquarters, clearly some workers considered that trust should be vested according to their rank and status rather than any hard-and-fast rules.

The payment of sick leave also raised questions about personal responsibility in relation to poor health. Although it was decided in 1871 ‘that in the classes entitled to the services of the Medical Officer, all diseases should have the attention whether venereal or otherwise’, nevertheless workers who could not be trusted to lead moral – and therefore healthy – lives often fell under suspicion.^91^^91^*ibid*., 182. Postmasters could draw attention to any cases which might require special notice, such as cases of ‘delinquency’, when an officer, whether by his own poor conduct or by intemperance, either impaired his own efficiency or disgraced the service – and therefore abused the privilege of receiving sick pay.^92^^92^*ibid*., 177–78. There were also situations in which for similar reasons workers might try to conceal the nature of their complaint, fearing that they might lose sick pay, or even be dismissed.^93^^93^*ibid*., 511. In this respect, venereal disease was seen as particularly problematic. Some managers considered it to be the result of a sufferer’s own irresponsibility and immorality and questioned whether under the circumstances the Post Office should be liable to pay sick leave. However, others were reluctant to withhold sick pay completely, for this might force sufferers to ‘resort to Quacks and thus seriously impair their health’.^94^^94^*ibid*., 175. It would also put the sick worker at ‘risk of his getting into debt and his becoming dishonest under the pressure of pecuniary embarrassments’.^95^^95^*ibid*., 175. Perhaps most importantly, it was believed that refusing sick pay could afford a motive for concealing the disease as long as possible and continuing to come on duty – ‘which would not be good for the service or the patient’.^96^^96^*ibid*., 175. The provision of sick pay, therefore, while on the one hand providing a temptation to malinger, also had the opposite effect of persuading workers who were genuinely sick to take leave and protect others. In an age when knowledge about the transmission of disease was still poorly developed, and where effective treatment was limited, measures that encouraged workers to quarantine themselves were prudent ways of stopping the spread of infection, however it was caused.

The system of medical oversight of sick leave clearly invoked a wide range of concerns relating to trust, status and personal responsibility. At its heart lay the financial penalties that sickness entailed, and it was important for the Post Office authorities to demonstrate that they were able to monitor and control the risks associated with unfit workers and spurious claims. Weeding out workers who were unlikely to be fit enough to last a lifetime at work was an important aspect of fiscal responsibility and there were attempts to achieve this at the initial recruitment stage. Thereafter, however, it was the responsibility of the doctors employed by the Post Office Medical Service to keep close track of sickness, even though this raised questions about status, professional trust, equity and personal responsibility. Demonstrating that the rising amount of sick leave identified in the records was genuine, and not the result of deliberate deception on the part of workers or lax monitoring practices, was of paramount concern to the Post Office and crucial to maintaining its reputation within government for fiscal responsibility.

## Conclusion

Trust lay at the heart of all the activities undertaken by the Post Office. As Patrick Joyce notes, through the materiality of its operations – its offices, post boxes and uniforms – and the behaviour of its workforce, the Post Office ‘became in time the object of a certain veneration, as something inherently British, efficient, unobtrusive and yet dependable’.^97^^97^Joyce, *op. cit*., 126.

The security of the mail helped to generate and reinforce confidence in good governance. When those operations were questioned, as happened in relation to the theft of mail or the monitoring of sick leave, issues of trust and responsibility were brought to the fore. The letters and parcels that were delivered in increasing numbers and frequency to middle-class households linked their well-being to the honesty and integrity of the humble postman. Businesses reliant on payment through the mail, such as mail order companies and friendly societies, which both increasingly came to depend on the transmission of money over long distances by way of postal orders, depended on the honesty of postal employees. Without that trust, the Post Office could not function, and without the Post Office, the progress of the nation and the integrity of the state was put in jeopardy. Trusting the post therefore had wider implications, not just for the well-being of middle-class Victorian families, who were its main customers, along with a growing number of businesses, but also for the confidence that was created in the ability of the British state to conduct its operations in honest and accountable ways.

In addition to this role, the Post Office also proved to be an important source of revenue for successive governments. It was by far the most profitable of all Civil Service departments and the Treasury therefore took a keen interest in its internal workings, especially in the granting of sick leave and pensions to the growing army of postal workers, both of which represented a drain on the state’s finances. From an early stage, the Post Office introduced a detailed system of medical checks to ensure and monitor workers’ health and their ongoing ability to work efficiently, conducted by its own doctors and paid for by the state. The system itself served to reassure the Post Office’s paymasters that responsible management and close monitoring of the workforce were in place in order to maintain financial control and remove inefficient workers at the earliest opportunity.

The questions of trust raised by the two different but complementary aspects of the Post Office’s operations also touch on a broader topic of concern for late nineteenth-century social commentators. In an increasingly urbanised and geographically separated world, social relationships between strangers, often mediated through the post, were becoming of ever greater importance. Towards the end of the century social observers, such as Emile Durkheim and Geog Simmel, questioned the bases of social solidarity, noting that trust between individuals and between the public and the state was of vital importance.^98^^98^B. Misztal, ‘The notion of trust in social theory’, *Policy, Organisation and Society*, 5 (1992), 6–15. Similar ideas about the significance of non-market exchange and social solidarity in relation to indigenous societies were also being explored by anthropologists such as Marcel Mauss. See *The Gift (Essai sur le don)*, first published in 1925. For a general introduction to different forms of trust see M. Sasaki (ed.), *Trust in Contemporary Society* (Leiden, 2019). See also C. Townley and J. Garfield, ‘Public trust’ in P. Mäkelä and C. Townley (eds), *Trust: Analytic and applied perspectives* (Amsterdam, 2013), 95–107. In his discussion about money, much like Mr Bushby the magistrate who had sat in judgement of Frank Foley’s misdemeanours, Simmel argued that ‘Without the general trust the people have in each other, society itself would disintegrate’.^99^^99^G. Simmel, *The Philosophy of Money* (London, 1990, first published 1900), 178. Matters of trust in the Post Office therefore speak to wider concerns common at the *fin de siècle*, not just about the social bonds between strangers but also between the public and the agencies of the state.

In this context, public confidence in the Post Office, which for many was the most benign representation of the state’s activities, was not merely about whether individual workers behaved with integrity but also about the competence of the institution itself to maintain a system of checks and balances that helped to ensure that the mail was delivered safely and efficiently by honest and healthy workers. Although the Post Office itself had a virtual monopoly on the delivery of mail, its reputation was nevertheless important and therefore its determination to seek out untrustworthy postal workers, whether through prosecutions of letter-stealers or the detection of malingerers, was a significant manifestation of sound and responsible management. The balance between public confidence and condemnation, however, was complex. It was not just a matter of detecting dishonest workers, whether they stole the mail or claimed sick leave for spurious reasons. It was also a matter of trust in the institution itself to behave in responsible ways, and this was – and still is – by no means easy to achieve.

